# Implication of Mesenchymal Stem Cells and Their Derivates for Osteochondral Regeneration

**DOI:** 10.3390/ijms23052490

**Published:** 2022-02-24

**Authors:** Veronika Smolinska, Michaela Debreova, Martina Culenova, Maria Csobonyeiova, Andrey Svec, Lubos Danisovic

**Affiliations:** 1 Faculty of Medicine, Institute of Medical Biology, Genetics and Clinical Genetics, Comenius University, Sasinkova 4, 811 08 Bratislava, Slovakia; smolinska7@uniba.sk (V.S.); martina.culenova@fmed.uniba.sk (M.C.); 2 National Institute of Rheumatic Diseases, Nabrezie I. Krasku 4, 921 12 Piestany, Slovakia; mdebreova@gmail.com; 3 Faculty of Medicine, Institute of Histology and Embryology, Comenius University, Sasinkova 4, 811 08 Bratislava, Slovakia; maria.csobonyeiova@fmed.uniba.sk; 4 1st Department of Orthopaedics and Traumatology, Faculty of Medicine, Comenius University, Pazitkova 4, 821 01 Bratislava, Slovakia; svec4@uniba.sk

**Keywords:** mesenchymal stem cells, exosomes, tissue engineering, osteochondral regeneration

## Abstract

Healing of articular cartilage defects presents a challenging issue, due to its regenerative shortcomings. Lacking vascularity and innervation of cartilage and low proliferative potential of chondrocytes are the main reasons for the limited healing potential of articular cartilage. Traditional reparative approaches are limited in their efficiency, hence there is a demand for novel reparative treatments. Mesenchymal stromal cells, preferred for clinical uses, can be readily derived from various sources and have been proven to have a therapeutic effect on cartilage and subchondral bone. Therefore, mesenchymal stromal cells, their derivates, and scaffolds have been utilized in research targeting osteochondral regeneration. The present review aims to comprehensively outline and discuss literature considering this topic published within last 5 years.

## 1. Introduction

Treatment of articular cartilage defects has proven to be a challenging subject in the field of regenerative medicine. Articular cartilage is known to have limited self-regeneration potential. It is complex, terminally differentiated, it lacks innervation and vasculature, and it is unable of clot formation, therefore it is not capable to onset a healing cascade [1,2]. Upon injury and/or cartilage lesion, mature chondrocytes do not produce sufficient extracellular matrix (ECM) and improper treatment often results in osteoarthritis (OA) development [3].

OA is an inflammatory and joint degenerative disease, which may be caused by trauma or auto-immune reactions, but also genetic predisposition presents an important factor in this disease. This debilitating disease affects the entire joint, causing degradation of articular surface and possibly deformity to subchondral bone. The degradational effects on the articular cartilage cause pain, malformation, and finally loss of function. Their progressive impact on articular cartilage is also irreversible. OA currently represents one of the leading causes of disability and affects up to 16% of the population aged 15 and over and was 22.9% in individuals aged 40 and over worldwide [4,5,6].

Another type of arthritis with an incidence of about 25 million patients worldwide is rheumatoid arthritis (RA). RA causes hyperplasticity of the synovium, producing a vast number of cytokines, chemokines, and autoantibodies, causing progressive degradation of joints, systematic complications, disability, and possibly leading to reduced life expectancy [7,8]. RA causes an autoreactivity process in the synovium and triggers chronical inflammation. The exact etiology of this disease is not fully understood. Research has proven that a complex cellular interplay (T cells, B cells, plasma cells, mast cells, stromal cells, synovial fibroblasts, and macrophages) is involved and soluble immune mediators are the major players in joint inflammation [9]. Currently, therapeutic options for RA treatment have inadequate results and numerous adverse effects causing additional issues. One of the presently available treatments is nonsteroidal anti-inflammatory drugs (NSAIDs), corticosteroids, and disease-modifying anti-arthritic drugs (DMARDs) such as methotrexate (MTX). It is frequently observed that a significant number of patients are non-responsive to these therapeutic strategies and their last resort is total knee arthroplasty (TKA), which is a very invasive surgery bearing high risks to patients’ health [10,11,12]. None of the traditional therapeutic approaches has shown satisfactory effects on the compromised joint, or has shown potential to restore chondral surface and prevent further decomposition of cartilage structure. In recent years, it became evident that novel therapeutic approaches need to be developed in order to provide less invasive and more effective strategies in regeneration of cartilage.

Mesenchymal stromal (stem) cells (MSCs) are known to not only have potential to differentiate into diverse cell lines depending on available niche, but also support their therapeutic potential via their paracrine activity. This demonstrates their wide possibilities of utilization in biological therapy for a vast number of diseases. The ability of MSCs to mediate immunomodulatory activities made them a reasonable candidate for novel treatment for autoimmune diseases, i.e., RA, systemic lupus erythematosus (SLE), Crohn’s disease (CD), and multiple sclerosis (MS) [13,14,15]. Their significant trophic effect is based on releasing cytokines, growth factors, and immunoregulatory proteins into their periphery [16,17]. MSCs used in therapy are derived from a number of sources. The most prevalent sources are umbilical cord, bone marrow, and adipose tissue [18]. The exact mechanism of MSCs’ effect on cartilage regeneration is still to be investigated. Furthermore, there is a need to consider the safety of MSC-based therapies, a universal protocol of administration, and finally identification of suitable patients for this particular therapy is required [19].

Thus, with the acquired knowledge of MSC paracrine activity being a principal function in regeneration, alternative “cell-free” approaches in tissue engineering have been developed [20]. MSC-exosomes have been shown to play a major role in MSC paracrine effect. They already confirmed their therapeutic effects by facilitating tissue repair in the heart, skin, and liver in a number of studies [21,22,23]. MSC-exosomes, commonly present in MSC secretome, are extracellular microvesicles (30–150 nm in diameter) made of lipid bilayer, incapsulating multiple cargos, capable of influencing cells and tissues through several signaling pathways without triggering an immune response [24,25]. Because of these representative features, recent studies have employed MSC-exosomes in cartilage regeneration and recognized their capacity to regulate chondrocyte homeostasis and coordinate subsequent regeneration processes via inducing chondrocyte proliferation, migration, differentiation, and matrix synthesis [26].

Another strategy showing great promise in regenerative medicine is additive manufacturing (AM). AM can be closely linked to MSC and/or MSC-exosome based therapies. AM techniques bring us numerous options in matrix- or scaffold-associated cartilage engineering, providing high precision complex structures, with remarkable mechanical properties, chemical composition, architecture, and porosity [27,28]. In recent years, researchers have targeted several possible scaffold materials, testing for their capacity to be printable, physiologically stable, and to provide cellular interactions when cells are seeded onto them. Combining three-dimensional (3D)-printed scaffolds with MSCs or MSC-derived exosomes provides promising tools that could offer patients cost-effective, custom-designed implants with the bio-functional properties of native cartilage [29,30,31].

Throughout the efforts of developing the ideal scaffold material, researchers have experienced challenges related to cartilage’s inability to degrade the artificial scaffold, due to its lower self-restorative capacity, avascularity, and hypocellularity [32]. Hence, the hypothesis of several research groups has shifted towards novel scaffold-free constructs. Employing bio 3D printers, omitting the scaffold portion, these groups are working towards development constructs made entirely of cell aggregates printed into 3D implants of desired shapes and sizes, in order to precisely match the osteochondral defect [33,34].

In this review, we focus on providing an updated overview of scientific publications in the field of regenerative medicine regarding treatment of OA and RA, exploiting MSCs and their derivates (exosomes), published within last 5 years. First, we address progress in clinical research involving different MSC sources, discussing approaches and findings of different research groups. We consider different ways of administration of MSCs to patients with osteochondral defects (intravenous infusion or intra-articular injection) and potentially shed light on the efficacy and safety aspects of this therapy, also in patients who have already been treated with DMARDs/NSAIDs. Furthermore, we provide information on various scaffold options utilized in delivering MSCs into cartilage defects of animal models. Afterwards, we consider novel scaffold-free strategies in MSC osteochondral therapy. Last but not least, we investigate MSC-derived exosomes and their utility in cartilage regeneration, considering different administration methods (Table 1).

**Table 1:** Overview of novel cell-based and cell-free OA and RA therapeutic strategies discussed in this review.

Trial Type	Human/Animal Model	Type of Cells/Product	Use of Scaffold	Way of Administration	Reference
clinical trial	human	autologous BMC	no	intra-articular injection	[35]
clinical trial	human	autologous BMSCs	no	intravenous infusion	[36]
clinical trial	human	autologous BMSCs	no	intravenous infusion	[37]
clinical trial	human	allogenic hUCB-MSCs	no	intravenous infusion	[38]
clinical trial	human	allogenic hUCB-MSCs	no	intravenous infusion	[39]
clinical trial	human	allogenic UC-MSCs	no	intravenous infusion	[40]
clinical trial	human	allogenic ADMSCs	no	intravenous infusion	[41]
clinical trial	human	allogenic ADMSCs	no	intravenous infusion	[42]
clinical trial	human	autologous ADMSCs	no	intra-articular injection	[43]
pre-clinical trial	rabbit	ADMSCs	infliximab-based hydrogel and 3DPMS	scaffold insert	[44]
pre-clinical trial	pig	ADMSCs spheroids	no	scaffold-free insert	[32]
pre-clinical trial	rabbit	ADMSCs spheroids	no	scaffold-free insert	[45]
pre-clinical trial	rat	hEMCS-exosomes	no	intra-articular injection	[46]
pre-clinical trial	rat	hEMCS-exosomes	no	intra-articular injection	[47]
pre-clinical trial	rabbit	U-MSC-exosomes	no	intra-articular injection	[26]
pre-clinical trial	rat	U-MSC-exosomes	no	intra-articular injection	[48]
pre-clinical trial	rat	U-MSC-exosomes	no	intra-articular injection	[49]
pre-clinical trial	rabbit	BMSC-exosomes	ECM/GelMA/exosome scaffold/bioink	scaffold insert	[50]
pre-clinical trial	rabbit	hWJMSC-exosomes	ACECM scaffold	scaffold insert	[51]

## 2. Literature Search Methodology

A search was performed (10 August 2021) of the PubMed/ Medline databases. Keywords related to MSC were combined with synonyms for osteochondral, cartilage, clinical trial, exosome, and scaffold. The search was restricted to the last 5 years and the English language. 

## 3. Results

### 3.1. MSC-Based Therapies in Clinical Trials

#### 3.1.1. Bone Marrow as Source of MSCs

Over the last few years, the focus of scientific activity has been towards investigating regenerative effects of both bone marrow concentrate (BMC) and bone marrow-derived MSCs (BMSCs) in cartilage repair. BMC, being a concentrate graft from bone marrow, aspirated usually from the iliac crest, contains a heterogenous cell population in which MSCs are present [52].

Heringou et al. [35] recently published a 15-year follow up to their original study, in which they treated OA patients who had to undertake total knee arthroplasty of both knees but chose not to have both surgeries done simultaneously. These patients, aged 65–90 years were offered an autologous BMC injection in the other knee (which was not undergoing TKA surgery), during the same anesthetic, on the same day. Patients were randomly assigned to two groups based on form of delivery of MSCs, either via intra-articular injection (IA group) or via implant in subchondral bone (SC group). Patients stopped taking inflammatory and/or analgesic drugs 3–4 weeks prior to the procedure; glucosamine was allowed for the patients who were previously using it. Post procedure, the patients were given analgesics (in relation with TKA), but no anti-inflammatory drugs. Follow-ups were performed at three and six months after surgery and then every year up until the recent 15-year follow up. In the next study, Heringou et al. [53] revealed several interesting findings from this study, mostly that in both SC group and IA group, injections of BMC led to significant pain relief. Conversion to TKA was postponed or avoided completely in the contra lateral joint of patients with bilateral osteoarthritis. Regarding the two cohorts, overall results have shown the subchondral injection to be more efficient in postponing TKA in the same grade of OA. The pain relief in case of the IA group generally did not last longer than 12 months, synovitis was not reduced, and lesions in subchondral bone were not decreased. Hence, many patients from the IA cohort eventually underwent TKA surgery for the particular knee. The most valuable finding is that the subchondral cell therapy treatment has the potential to become a primary treatment of OA, since this study presents long-term benefits (15 years) of the treatment and its ability to postpone, and even in some cases completely avoid TKA in some patients. This approach utilizes rather low concentrations of the MSC in BMC graft, and it is not possible to demonstrate the optimal MSC concentration, due to the heterogenous character of bone marrow graft. Consequently, we could consider this a factor of improvement of this technique, since the MSC number in BMC decreases relative to the age of the patient [54]. In order to move away from the heterogeneity of BMC grafts from the iliac crest, many studies are gravitating towards the route of cultured MSCs. By isolating, characterizing, and expanding the stem cell populations, we can overcome low stem cell yield and donor side morbidity limit [55].

Shadmanfar’s group applied these cultured BMSCs in their research into RA patients’ knees via intra-articular injections. In their study, they used approximately 40 million autologous MSCs. This study is the first triple-blind placebo-controlled clinical trial targeting the safety and tolerability of BMSCs therapy in RA-involved knees. The group was also able to achieve early clinical efficacy of the therapy. Patients enrolled in the study, who were 18–65 years old (mean age average was 50 years), were allowed continue taking DMARDS, but not allowed NSAIDs. Patients were randomly assigned to either receive MSC treatment or placebo (sterile saline), received the treatment once, and returned for check-up after 1, 3, 6, and 12 months in order to record the safety and efficacy of the treatment. Bone marrow was aspirated from iliac crest of each patient, and BMSCs were isolated and then cultured for 7–10 days, and then injected into the patient’s knee (if assigned to MSC group). In this study, no adverse effects were recorded during the injection, nor any of the follow ups. The MSC-treated group showed remarkable improvement in pain relief already 1 month post injection and was able to maintain this effect until the last follow up (at 12 months). There was a significant decrease in the Western Ontario and McMaster Universities Arthritis Index (WOMAC) scores for the MSC group (of −16.5 ± 13.5) vs. the placebo group (−6.7 ± 13.6), which indicates a major decrease in joint pain. Finally, importantly, unlike the placebo-treated subjects, the visual analogue scale (VAS) assessment proved the effectiveness of MSC treatment by decreasing knee pain by 50% at the last follow up. The study claims BMSCs’ intra-articular injection into patient RA-involved knee to be safe, well tolerated, and feasible at the employed dose and study design. The results also suggest a clinical benefit of the study but this is to be further researched with a larger number of RA patients with knee involvement [36]. 

Recently, a very unique study concerning immune-related genes in autoimmune diseases and their expression was published by Ghoryani and his colleagues. In this clinical trial, refractory RA patients (of 44 ± 7:50 years), who were receiving the maximum approved dose of conventional DMARDs, were recruited. The patients intravenously received autologous MSCs isolated from the bone marrow. Cells used in this study were harvested and cultured, developing more homogenous culture of BMSCs. Each patient received one dose of MSCs, in which the number of cells varied based on subject’s body weight (BMSCs, 1 × 10^6^ cells per kg). Immunological factors were obtained from patients’ peripheral blood mononuclear cells (PBMCs) and evaluated at 1, 6, and 12 months. The results presented by Ghorvani’s group showed substantial increase in IL-10 and transforming growth factor-beta 1 (TGF-β1) levels, both of which are two major cytokines of Tregs. Additionally, the study showed increased levels of forkhead box P3 (FOXP3), a unique Treg transcription factor, at the end of the intervention (at 12-month check-up). This actively demonstrates that the BMSCs had immunoregulatory effect on regulatory T cells, possibly differentiating T lymphocytes from Tregs. The scientific group discovered via correlation analysis a negative relationship between levels of IL-4 and the Disease Activity Score-28 for Rheumatoid Arthritis (DAS28-ESR). All the previously stated data were supported by demographic data, showing a considerably lower DAS28-ESR compared to before the treatment, throughout the whole study. None of the patients reported any adverse events at any of the follow-ups during the study. In future research, increase of MSCs and/or repetitive treatment with MSCs will be looked into [37].

#### 3.1.2. Umbilical Cord as Source of MSC

Human umbilical cord became a prominent source of MSC in regenerative medicine. Human umbilical cord blood-derived MSCs (hUCB-MSCs) possess several advantageous properties compared to BMSCs, including accessibility, increased proliferation, and decreased immunogenicity, which makes them an interesting option for RA treatment. Their immunoregulatory traits make them promising allogenic source of MSCs to be used in therapy. Although their repair mechanisms in RA therapy have not been fully exposed, they are recognized for their self-restorative properties and multipotential differentiation ability [56]. To this day, there is a lack of comprehensive reports concerning clinical benefits of hUCB-MSCs in the treatment of RA, as well as safety evaluation, and possible cartilage repair mechanism. 

Wang and colleagues conducted a cohort recruiting active RA patients with inadequate response to DMARDs. They divided these patients into two groups; the first group received DMARDs with hUCB-MSCs, and the second one DMARDs with medium without hUCB-MSCs. The DMARDs + hUCB-MSCs group intravenously received a small dose of DMARDs along with 4 × 10^7^ of hUCB-MSCs in stem cell solvent. The DMARDs + medium without hUCB-MSCs group received a small dose of DMARDs with stem cell solvent without hUCB-MSCs, also via intravenous infusion. Additionally, subjects in the DMARDs + hUCB-MSCs cohort received more than one treatment and therefore were divided into 3 groups according to different intervals after the first treatment. Group 1 received the second treatment after 3 months, group 2 after 6 months, and group 3 after 8 months. In order to assess the safety of the treatment, patients’ physical health, liver, and kidney function was evaluated along with hematological and biochemical testing, urine analysis, chest radiography, and electrocardiograph (ECG) being performed before and after the treatment (for both treated and control group). No major abnormal findings related to adverse effects were observed in the study. Clinical effects suggest that DMARDs + hUCB-MSCs administration resulted in long-term mitigation of disease activity of refractory RA [38]. It is believed the clinical benefits were linked to the decreased expression levels of inflammatory cytokines and chemokines, the increase in regulatory T cells in peripheral blood, and the upregulation of IL-4-producing Th2 cells [57]. Wang and his group suggest the major potential mechanism behind positive effects of hUCB-MSCs are anti-inflammatory affects along with the improved immune-modulation and the induced immune-tolerance. Rapid joint pain relief and alleviation of joint swelling within 12 h post-treatment was reported, and maintained throughout the whole study. The second cycle delivered even better clinical benefits, and over all resulted in better quality of life for the treated patients [38]. The data suggest that patients infused with MSCs that are HLA haploidentical or completely HLA mismatched with the stem cell donor and recipient show no difference in clinical effects and have no immunological memory to the infused MSCs [58]. The core message of this study is that RA patients who were previously nonresponsive to traditional medication treatment received significant improvements, including symptom alleviation and cytokines decrease, when treated with hUCB-MSCs, and these positive effect of the MSC therapy were prolonged and stabilized via repetitive treatment [38].

The first phase Ia clinical trial extending their preclinical research on hUCB-MSCs safety and tolerability in patients with RA was published by E. Park et al. Treatment was delivered via single intravenous infusion of cultured hUCB-MSCs. This was an open-label, dose-escalation study and the study subjects had moderate RA and were on a stable dose of methotrexate (MTX) for at least 12 weeks before being enrolled into the study [39]. Throughout the study, patients maintained their regimen of corticosteroids; coincidentally none of the patients previously received DMARDs. Patients were divided into 3 groups, of which the first cluster received 30 min infusion of lowest dose, with cell number of 2.5 × 10^7^ hUCB-MSCs. When no dose-limited adverse events arose, the next group received a dose of 5 × 10^7^ cells. Safety and tolerability were assessed for this cohort before moving to the final patient cluster, who received the highest dose of 1 × 10^8^ of hUCB-MSCs. Safety and tolerability were measured (hematological and biochemical tests, urine analysis, chest radiography, and ECG) after 24 h, 72 h, 1 week, and 4 weeks following the infusion. Preliminary efficacy assessment (via DAS28, a pain visual analog scale (VAS), and health assessment questionnaire (HAQ)) was obtained after 4 weeks. In this study, no adverse events nor dose-limiting toxicity (DLT) was reported. Although assessing the clinical benefits of hUCB-MSC treatment was not the primary objective in this trial, Park and colleagues reported a decrease in DAS28 at week 4 after the treatment, and a decrease in IL-1β, IL-6, IL-8, and TNF-α levels were measured at 24 h in the highest dose cohort (1 × 10^8^ cells). Additionally, this study reported a significant increase in IL-10 levels, in a cluster treated with 5 × 10^7^ cells after 24 h. For the future trials, this research group plans to investigate long-term DLT and safety, including a placebo group, as well as evaluate the safety of repetitive infusions, including patients previously treated with DMARDs, and the assessment of clinical outcomes utilizing imaging methods [39].

A recent study by Wang et al. [40] utilized umbilical cord tissue-derived MSCs (UC-MSCs) and demonstrated its long-term safety and efficacy in RA therapy. The data presented in this study proved stable clinical outcomes for 3 years post single dose treatment. The data presented showed that combination of DMARDs with cultured UC-MSCs therapy was safe, and drastically improved quality of life of RA patients included in this trial. They had previously reported safety and efficacy of this treatment for up to 8 months, and now have proven that the DMARDs + UC-MSCs treatment alleviated RA symptoms, reduced HAQ, and DAS28 scores long-term [40]. Furthermore, the levels of inflammatory and/or RA serological makers significantly decreased, and were maintained for 3 years in comparison to pre-treatment. All patients given DMARDs with UC-MSCs reported rapid remission in disease activity and had improvements in diet, sleep, and physical strength compared to control group, who experienced no such improvements. In this study, they presented two particularly extraordinary clinical outcomes of two patients, who experienced remission nearly 6 months post-treatment, and have since maintained these positive effects, which enables them free movement and a life free of pain, joint swelling, and deformity [40]. The same group, provide evidence that UC-MSCs based therapy seem to be efficient and safe to be used in clinical practice [59]. 

#### 3.1.3. Adipose Tissue as Source of MSC

Lately, an alternative source of MSCs has been employed in clinical research of RA; adipose-derived mesenchymal stem cells (ADMSCs) propose an easy access with minimally invasive procedure with multiple collection sites [60,61]. ADMSCs have proven to be capable of multilineage differentiation, with high proliferative potential and surface proteins, that make them a suitable candidate for cell-based therapeutic strategies [62].

The research team of Álvaro-Gracia published results of the first multicenter, dose escalation, randomized, single-blind, placebo-controlled, phase Ib/IIa clinical trial with active refractory RA patients, intravenously treated with allogenic ADMSCs. The primary goal of this study was to assess the safety of allogenic ADMSCs intravenous infusions, and find DLT [41,63]. The secondary purpose was double-blinded preliminary efficacy evaluation. Subjects enrolled in this study were previously unsuccessfully treated with at least one to two standard anti-RA non-biological treatments. The study included a wash-out period without treatment before the trial started. Patients were randomly divided into 4 cohorts, according to the number of cells they received—cohort A: 1 million/kg, cohort B: 2 million/kg, cohort C: 4 million/kg, or placebo: receiving Ringer’s lactate solution. The treatment was administered via three intravenous infusions at days 1, 8, and 15, and follow-up visits were conducted at weeks 1, 2, and 3, and at months 1, 2, 3, 4, 5, and 6 [41]. In this study, the researchers decided to utilize allogeneic ADMSCs, due to their availability in the cell bank, which makes them readily available for usage. Cells were originally obtained from lipoaspirates and were tested for viability, population doublings, morphology, potency, identity, purity, sterility, and genetic stability, among other quality controls. The presented data suggest that no treatment-related toxicity occurred and the therapy resulted in an overall favorable safety profile. No venous thrombosis or pulmonary thromboembolism arose, nor did any life-threatening events or deaths occur. One DLT was reported in a subject from cohort A at day 8 after the 2nd infusion, who suffered lacunar stroke, which was deemed as likely related since no other apparent causes were found. The purpose of this study was mainly to investigate toxicity and safety of this therapy, although the efficacy data provided in this trial showed a better response in comparison to the placebo group. Nonetheless, assessment of clinical efficacy outcomes must be cautiously interpreted, since the study was not designed towards efficacy evaluation [41].

Mallinson’s research group has published data from a distinctive study with the main objective to stratify highly refractory RA patients receiving MSC therapy, in order to investigate biomarkers associated with ADMSCs therapy response in RA patients [42,64]. For this trial, they selected RA patients who were previously intravenously treated with MSC therapy, and either responded to the therapy (responders), or did not respond to the MSC treatment (non-responders). RNA from pre-treatment plasma samples from both responders and non-responders were analyzed via circulating miRNA microarrays. These miRNA biomarkers were further investigated so as to accurately evaluate relative expression between two patient groups and 10 most significantly differentiated miRNA biomarkers were selected [42]. Based on statistical significance, 3 final candidates were established—miRNA biomarkers miR- 26b-5p and miR-495-3p were recognized to be significantly upregulated in the responder group, and miR-487b-3p came very close to being significantly upregulated. The study claimed that the three targeted miRNA biomarkers present potential in discrimination between cell-based therapy responders vs. non-responders, which makes them a crucial element in the success of these pioneering therapeutic approaches. Although the presented results seem promising, there is a need for a larger sample size and further investigation of the three miRNA biomarker candidates [42].

Lee et al. published results from their most recent phase IIb, double-blinded, and placebo-controlled clinical trial, assessing the safety and efficacy of intra-articular autologous ADMSC injections in patients with knee OA [43,65]. Patients were blindly assigned to ADMSCs injection (MSC group) or normal saline injection (control group). The MSC cohort received a high dose of cells (1 × 10^8^ cells) and patients’ activity was not restricted, allowed full weight-bearing post-treatment. Enrolled subjects, between 18 to 75 years old, had OA of the knee joint. All anti-OA medication was discontinued for 2 weeks before the treatment. Cells were harvested via lipoaspiration from the participants and culture-expanded. Cell number, viability, purity (CD31, CD34, CD45), identity (CD 73, CD 90), sterility (bacterial and fungal), and endotoxin and mycoplasma contamination was evaluated before intra-articular administration [43,66]. Efficacy and safety were inspected at 1, 3, and 6 month follow ups. In this report, Lee and his colleagues showcased significant changes in cartilage defect after single treatment of autologous ADMSCs. This one-step treatment resulted in 55% reduction in the WOMAC total score, 59% in the WOMAC pain score, 54% in the WOMAC stiffness score, and 54% in the WOMAC physical function score at 6 months post-treatment. This study was lacking in sample size, hence the group suggests a larger group and longer follow-up is needed in the next trial [43].

It is difficult to fairly assess differences between particular MSC sources and discuss whether UC-MSCs, ADMSCs, or BMSCs are superior to the others. The variances such as study design, cell type, rehabilitation protocols, and adjunct therapy hinder the possibility to statistically evaluate the clinical studies [67,68].

Furthermore, the utilization of allogeneic vs. autologous MSCs in treatment is to be investigated more deeply in order to compare their therapeutic potential. Autologous MSCs are believed to be safer, since they do not initiate an immune reaction. Nevertheless, the donor site morbidity might be a downfall of the autologous MSCs compared to the allogeneic MSCs. However, tumorigenesis, disease transmission, and possible host immune rejection still remain a concern when dealing with allogeneic MSCs and must be studied in the future [69,70]. 

### 3.2. Scaffolds and MSCs Combined

Presently, it has been hypothesized that combining MSCs with a proper delivery system would enable better regeneration, particularly with the support of bio-functional scaffolds, that would offer the benefit of structure and properties related to the native tissue. Tissue engineering now relies on additive manufacturing (AM) and newly developed non-degradable and biodegradable materials. These are supposed to functionally support MSC as matrix/scaffold where cells are seeded onto. Properties such as mechanical strength, porosity, bioactivity, printability, stability, biological characteristics, and many more are limiting factors that narrow down the choice of synthetic polymers suitable for AM [71,72,73]. Scaffolds are very complex and are constructed with very high precision, which allows creating patient-specific tissue implants. Various biomaterials may be produced, including thermoplastics which are currently investigated for printing 3D bone grafts [29]. Recently, researchers started to combine co-polymers such as poly(ethylene oxide terephthalate) (PEOT)/poly(butylene terephthalate) (PBT) (PEOT/PBT) with a range of bioactive nanomaterials in order to achieve better outcomes [74,75,76]. It has been presented that nanosilicates, when mixed in with co-polymers, provide remarkable biocompatibility, do not trigger immune reaction, and due to their surface charge can be homogenously distributed. Additionally, there are data proving the upregulation of osteogenic markers in human MSCs (hMSCs) when treated with nanosilicates [77,78,79,80,81,82].

Likewise, hydrogels have shown their potential specifically due to their osteochondral regenerative qualities. Hydrogels, containing hydrophilic chains in an aqueous microenvironment, possess qualities such as biocompatibility, aqueous nature, variability in mechanical properties, and features that demonstrate their promise in the field of regenerative medicine [83,84]. 

Hydrogels bring countless possibilities which inspired researchers from Cross’ team to engage in creating two-dimensional (2D) nanocomposite gradient hydrogels. Their goal was to introduce an approach with a high reproducibility rate in order to consistently fabricate gradient hydrogel consisting of two natural polymers, gelatin and kappa carrageenan, with the addition of nanosilicates. The data presented in their manuscript showed the ability to achieve gradient in pore size and mechanical properties, indicating successful manufacturing. The group was able to manifest how the presence of such gradient-influenced morphology of encapsulated MSCs directly, hence possibly control cell fate via the gradient and nanosilicates incorporation [78].

**Figure 1:**
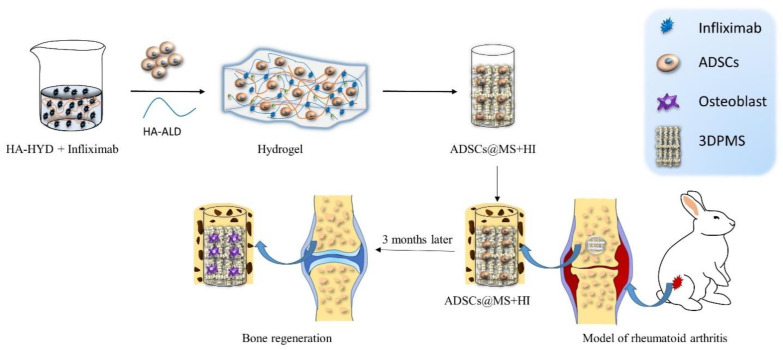
Schematic illustration of assembly of self-healing infliximab-based hydrogels combined with 3DPMS assembly with objective to deliver ADSCs supporting RA management.

Hydrogels have attracted considerable interest from Zhao et al. [44] and encouraged them to incorporate therapeutic antibodies, specifically infliximab, into hydrogel which was then used to encapsulate ADMSCs. They fabricated infliximab-based, self-healing hydrogel for regulating the hostile microenvironment of the RA site. The presented data showed that infliximab-based hydrogel enhanced the survival, engraftment, and function of ADMSCs. The designed hydrogel consisted of HYD-modified HA (HA-HYD) and ALD-modified HA (HA-ALD) solution, and optimized concentration of infliximab. This hydrogel completely degraded within 30 days, while steadily releasing infliximab into the damaged cartilage of the rabbit model (Figure 1).

The infliximab-based hydrogel was then combined with 3D printed porous metal scaffolds (3DPMS), which were previously developed by Zhao et al. [85], and inserted into the osteochondral defect of an animal model. The presented results act as evidence that the viability, proliferation, and osteogenic differentiation capacity of encapsulated ADMSCs were maintained even under RA conditions. Down-regulation of inflammatory cytokines, induced osteogenesis, cartilage rebuilt, and improved bone repair detected in rabbit model after 3 months confirm the potential of employing antirheumatic drugs to construct hydrogels for stem cell-based therapies of RA [44].

### 3.3. MSC-Based Scaffold-Free Tissue Engineering

The main challenges faced with scaffold-based cell therapies are that the actual scaffold insert may actually hinder cartilage regeneration. Scaffolds remaining at the implanted sites for long time periods promote fibrous cartilage generation. Articular cartilage is specific for its low restorative capacity, avascularity, and hypocellularity; hence, it has limited abilities to deteriorate foreign scaffolds. Furthermore, another major concern regarding scaffold implants is immunogenicity and long-term safety of the insert and its degradation products. At the moment, there is no general scaffold material that is certain to be ideal for osteochondral regeneration [86,87,88]. Therefore, scientists invested into developing 3D implants created entirely out of cells. One method by which these constructs are fabricated is by manually stacking cell aggregates (spheroids) into cylindrical molds. The second, more refined method, is bio 3D printing. Bio 3D printers introduce high levels of customization to constructs, with predefined shapes, densities, and spheroid distribution [89,90].

Recently, D. Murata and his team carried out a number of studies regarding the topic of scaffold-free 3D constructs built from ADMSCs, utilized for osteochondral regeneration in animal models [45,91]. The study, published in 2018, presented the histopathological results of regenerative potential of 3D inserts, constructed out of autologous ADMSCs [32]. These inserts were implanted into the osteochondral defect of a pig model. The regeneration of articular cartilage and subchondral bone was assessed after 6 and 12 months. Three-dimensional implants were prepared by placing approximately 770 spheroids into a cylindrical mold, allowing the spheroids to fuse with one another. Afterwards, the mold was carefully removed and the cylindrically shaped insert was ready for autologous implantation. Two cylindrical osteochondral defects were created in the patello-femoral groove of a pig; a columnar 3D construct composed of ADMSCs spheroids was autografted into one of the two defects. The second defect was not implanted into, therefore creating an implanted and a control group in one knee of a pig. The presented results showcase the scaffold-free 3D ADMSC implants induced hyaline cartilage and subchondral bone regeneration. At 12 month follow up, there was a visible difference in uniformity between the implanted and the control site. In the implanted site, the boundary between implant and normal cartilage was not clearly visible, and the defect appeared uniform and smooth. Histologic scores, cellular morphology, Safranin-O staining, and chondrocyte clustering in the implanted defects were substantially superior to the control defects without an implant. The evidence suggests the predominant tissue in treated defects was mainly fibrocartilage, while the control defect appeared to be made up with fibrous granulation tissue. The analysis showed the construct differentiated into two different tissues, based on the environment. The surface layer of cells differentiated into cartilage, while the deeper layer was able to differentiate into bone. In order to accurately observe and evaluate regeneration and degree of osteochondral reconstruction, researchers planned to obtain computer tomography (CT) and MRI data in their future studies [32].

The following study carried out by D. Murata et al. [45] in 2020 explored osteochondral healing of a knee defect in a rabbit model after 3 months utilizing CT and MRI. In this instance, they employed bio 3D printed tubular tissues, with approximately 960 ADMSCs spheroids per construct. They claimed to overcome viability issues of 3D structures, since viability in the construct was reported to be more than 80% compared with previous studies on spheroid culture systems [92]. The ADMSCs aggregates were proven to have differential potential. Animals were randomly assigned to two cohorts; the first group received an autologous implant, the second one was left untreated and therefore did not receive an implant in the defect. Follow-up was set to 12 weeks post-implantation. Overall, the presented data showed remarkable construct-facilitated repair of the defect (nearly to the extent of surrounding osteochondral tissue), in comparison to open and barely healed defects in the control group. Regarding the subchondral bone regeneration, researchers claimed that a 3-month time period might have not been sufficient for the novel cartilage tissue to undergo osteochondral ossification. Hence, future studies will incorporate more follow-up time points with duration up to 24 weeks post-treatment [45].

Scientific evidence presented in the manuscripts discussed above suggest that the scaffold-free approach is a promising tool for osteochondral regeneration omitting tedious testing of artificial scaffold materials utilized for MSC delivery. Nevertheless, it would be helpful to execute comparative studies to compare scaffold-based and scaffold-free MSC treatment methods and their suitability for osteochondral regeneration. Finally, it must be verified whether these methods can be safely and successfully extrapolated into humans.

### 3.4. MSC-Exosomes

Despite the primary hypothesis that MSC-based therapeutic approaches facilitate cartilage repair by replacing damaged tissue via their ability to differentiate, it is now generally established that the actual therapeutic mechanism lays in their secretory activities. Several technical limitations occurred with these cellular approaches; for instance, labor-intensive and time-consuming cell expansion, dedifferentiation during cell expansion, inconsistency in large-scale production, reduction of intrinsic activity upon administration, and last but not least pulmonary embolism associated with MSC-based cell therapy [93,94,95,96,97].

**Figure 2:**
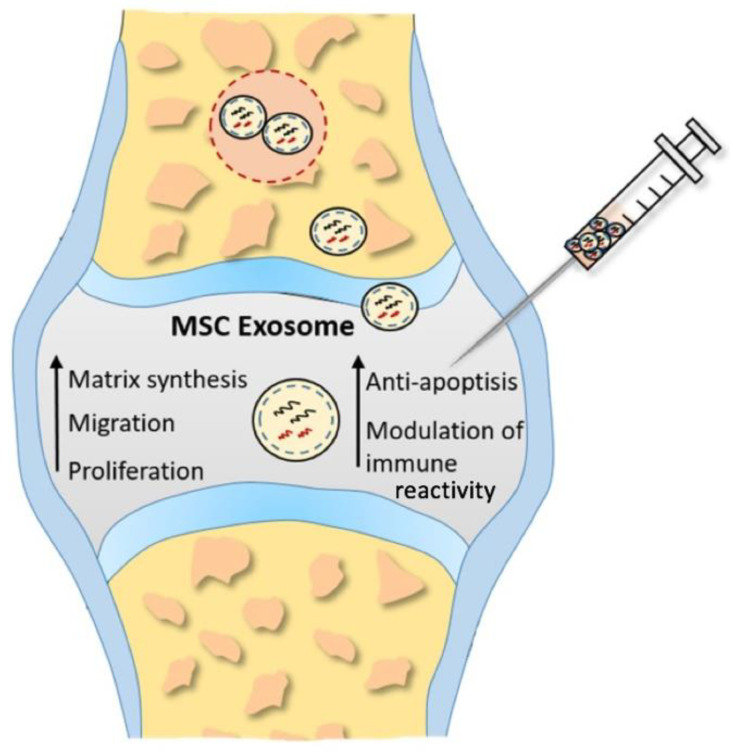
Proposed underlying therapeutic mechanisms of MSC exosomes in cartilage restoration. Enhancing proliferation, migration, and matrix synthesis, as well as attenuating apoptosis and modulating immune reactivity, inducted by MSC exosomes promotes cartilage repair and regeneration.

Hence, the attention is directed towards MSC-exosomes, nanosized extracellular vesicles, acting as natural carriers of therapeutic molecules (Figure 2). The theory is that exosomes could replace conventional cell-based treatments, overcoming the limitations of cell-based methods mentioned earlier [25,98].

To demonstrate the therapeutic potential of exosomes, S. Zhang et al. [46] executed a proof-of-concept study. Their evidence supports the idea that this cell-free therapeutic strategy greatly encourages regeneration of hyaline cartilage and underlying subchondral bone. The study was executed in immunocompetent rat models with osteochondral defects, which were intra-articularly treated with human embryonic MSC-derived exosomes (hEMCS-exosomes) once a week for 12 weeks. The final results after 12 weeks showed major differences in regeneration between the group treated with hEMCS-exosomes versus the control group treated with PBS. No adverse inflammatory responses were observed in any of the animals, which is a good indication for possible application of MSC-exosomes for allogeneic use in human patients.

Furthermore, Zhang’s research group published a manuscript reporting that MSC-exosomes directly influenced migration, proliferation, apoptosis, and matrix synthesis of chondrocytes in vitro. Through their diverse proteomic and RNA cargo, MSC-exosomes mediate cartilage repair by a multi-faceted mechanism (i.e., induction of AKT/ERK signaling), which increases the expression of genes associated with proliferation (PCNA and FGF-2), and anti-apoptosis (Survivin and Bcl-2). In future studies, it will be necessary to determine a therapeutic window for fewer MSC exosome injections, therefore minimal effective dose, as well as investigation in larger animal models [47].

Lately, intense efforts have been made to establish the most effective large-scale exosome production system [99,100]. The commercial hollow-fiber bioreactor system was the standard mechanism for production of exosomes, by conventional 2D culture [101]. Yan and his research team investigated the differences in cellular mechanisms, processes, and chondroprotective properties of exosomes produced by 2D culture and 3D culture of U-MSCs in hollow-fiber bioreactor. In this study, cells were seeded into cylindrical fibers in order to simulate 3D culture in the bioreactor. As a consequence, there was a remarkable improvement in yield in 3D culture (7.5-fold higher than in 2D) and biological function in vivo (rabbit osteochondral defect model, intra-articularly treated weekly for 4 weeks) of the 3D exosomes showed superior therapeutic effect than 2D exosomes. Although 3D exosomes dominated 2D exosomes in in vitro tests, as well as curative effects, the mechanism behind this is still unclear and will be looked into in future studies, focusing on comparative proteomic and RNA-seq analyses [26].

Later on, several groups published studies where they decided to explore improving the scalable acquisition method of MSC-exosomes by employing mechanical stimuli for exosome generation [49,102,103]. They utilized a rotary cell culture system (RCCS) for production of U-MSC-exosomes. The determined optimal rotation rate was 36 rpm. Three types of exosomes were produced: exosomes from U-MSCs cultured without RCCS mechanical stimulation (N-Exos); exosomes from U-MSCs transfected with siRNA H19 in a mechanically stimulated environment (si-Exos); and exosomes from U-MSCs with mechanical stimulation in RCCS (S-Exos). The presented data confirm S-Exos to be superior in quality of MSC secretome, hence inducing cell proliferation and matrix synthesis, while inhibiting cell apoptosis more effectively. The in vivo experiments were executed on cartilage defected rat models, who were treated with intra-articular injection of either S-Exos or si-Exos once a week. The advantage of S-Exos was finally visible at the 8 week follow-up, with a significant difference in repair between this group and si-Exos, and the control group. Additionally, these results suggest that mechanically stimulated U-MSCs produced exosomes with an increase in H19 expression. The study proposes that LncRNA H19, involved in stem cell differentiation, embryonic growth, and tumorigenesis, may promote proliferative and anti-apoptotic processes in chondrocytes. Since it was presented here that the interference against H19 in U-MSCs remarkably weakens the efficacy of exosomes, the researchers claim the LncRNA H19 promotes chondral damage repair [49].

This hypothesis was then verified in their pioneering paper which revealed ability of U-MSC-exosomes to transfer lncRNA H19 to chondrocytes, suggesting miR-29b-3p/FoxO3 as the downstream signaling pathway [48]. They claim, that upon delivery of lncRNA H19 to the chondrocyte, it competitively binds to miR-29b-3p, resulting in FoxO3 regression, thereby causing boosted chondrocyte migration and matrix synthesis and mitigated apoptosis and senescence. Hence, the study indicates a possible therapeutic target for posttraumatic focal cartilage deficiencies [49].

In order to fully benefit from the osteochondral regenerative capacities of MSC-derived exosomes, there is still a lot that is ambiguous. Mainly, fundamental composition analysis and related therapeutic potential should be studied in more depth. Furthermore, administration methods should be reconsidered so as to move away from the conventional intra-articular injection delivery.

### 3.5. MSC-Exosomes and Scaffolds

Recent studies focusing on developing cell-free approaches in osteochondral defect treatment invested into the design and fabrication of hydrogels and scaffolds for exosome delivery. Developing such a delivery system for exosomes to facilitate repair of osteochondral defects, one must keep in mind several requirements that this system must meet, namely, cell recruitment ability, since chondrocyte migration has been shown to be one of the major processes in cartilage healing [104,105].

It has been previously demonstrated that decellularized natural ECM might encourage cell recruitment, infiltration, and differentiation while minimizing immunologic reactions [106,107]. A study presented by Visser et al. [108] reported that ECM supports endochondral ossification.

It is necessary for scaffolding, if destined to be utilized in cartilage regeneration, to also fulfill the requirement for adequate robustness, due to joint load-bearing function [109]. Combination of decellularized cartilage ECM (porcine origin), gelatin methacrylate (GelMA) hydrogel, and autologous BMSC-exosomes exhibited proper mechanical attributes to be utilized in load-bearing tissue, thus osteochondral tissue. Chen et al. achieved sturdiness by photo-crosslinked printing of this bioink into radially oriented scaffold, using desktop-stereolithography (SLA) technology [50]. They claim that this low-cost technique allows for a precise layer-by-layer assembly and has the capacity to effectively retain exosomes for up to 2 weeks. The data presented in the study suggest that the ECM/GelMA/exosome scaffolds enhanced chondrocyte migration into the defect site, polarized the synovial macrophage response towards the desirable M2 phenotype, and last but not least was effective in restoring cartilage mitochondrial dysfunction in chondrocytes [50,110]. They proved the MSC-exosomes released from the scaffold were successfully internalized by the chondrocytes and were able to enrich various regions of their mitochondria. Recommendations for future research include to target proteins relevant for this recovery. Rabbit and rat animal osteochondral defect models in this study were surgically treated once, instead of periodical injections, as in the MSCs/MSC-exosomes studies [50].

In another study, porcine articular cartilage was decellularized and used to prepare porous, vertically oriented “acellular cartilage extracellular matrix” (ACECM) scaffold, which was then placed in a cylindrical mold to achieve the desired shape [51]. In the study by S. Jiang et al., the ACECM scaffold was combined with Human Wharton’s Jelly MSCs-derived exosomes (hWJMSC-exosomes) [51,111,112]. This “cell-free” tissue engineering method was tested to see if it could achieve osteochondral regeneration. Firstly, the data from in vitro studies showed boosted migration, proliferation of BMSCs, and enhanced proliferation of chondrocytes induced by hWJMSC-exosomes. Afterwards, the research group applied a rat osteochondral defect model to explore these results in vivo. Rats received weekly intra-articular injections of either exosome suspension (exosome group) or PBS (control group) for 10 and 20 days. Levels of inflammation and macrophage polarization were evaluated by immunohistochemical staining, which showed that exosome injection promoted towards M2 phenotype in macrophages, hence increased IL-10 levels, thereby inhibiting inflammatory processes [51]. The presented results showed only a slight increase in the number of migrated BMSCs in the defect of the exosome group, compared to the control group. Subsequently, to evaluate the reparative effect of the ACECM scaffold, rabbit osteochondral defect models were divided into 4 groups: PBS group, PBS + scaffolds (PBS + S), MSC-exosome group (Exo), and MSC-exosome + scaffold group (Exo + S). This study design also included a sham group. The scaffold was inserted into the defect surgically and rabbits were administered PBS/exosome injections every 7 days for 3 to 6 months. The overall results, collected during a 6-month follow-up, indicated the best repair appeared in the Exo + S group, revealing high content of type II collagen in newly formed cartilage tissue, almost reaching levels of normal cartilage of the rabbits from the sham group. The group treated with the combination of ACECM scaffold and MSC-exosomes displayed enhancement in cartilage and subchondral bone regeneration, which provides affirmative proof that this synergic duo may be a promising tissue engineering strategy in osteochondral regeneration. Finally, the study presented miRNA sequencing and analysis data that identified 20 miRNAs regulating joint cavity microenvironment. This will be helpful to determine key components and signaling pathways in hWJMSC-exosome-induced osteochondral regeneration and cartilage regeneration regulatory targets in the future. Optimal dosing experiments shall be executed in order to establish the ideal dosage regimen [51].

## 4. Discussion

This analysis of literature allowed us to underline interesting findings in MSC-based osteochondral therapy strategies within the last 5 years that deserve to be discussed. Firstly, we reviewed clinical trials utilizing mesenchymal stem cells derived from bone marrow. Bone marrow may be a source of heterogenic bone marrow concentrate (BMC), a patient material which is not cultured, or bone marrow-derived MSCs, which are applied upon culture expansion, granting more homogenic character. The patients received autologous MSC treatment either via intra-articular injection or intravenous infusion and reported clinical benefits including pain relief, reduced joint stiffness, and increased maneuverability [36,37,53]. Additionally, we presented a 15-year follow-up study which confirmed the long-term effects of this therapy approach and the ability to postpone or completely avoid TKA surgery [53]. Another prominent MSC source covered in this review was the human umbilical cord. This allogenic source, either used by itself or combined with DMARDs treatment, successfully alleviated disease symptoms with a long-term effect on the disease progression. Recent studies suggest that the hUCB-MSCs are advantageous in cartilage repair compared to BMSCs, due to their improved proliferation, accessibility, and lack of immunogenicity [38,39,40]. Last but not least, the most accessible source with least donor site morbidity are ADMSCs, which are currently widely researched for future osteochondral therapies. Stem cells derived from adipose tissue display both allogeneic and autologous potential in the most recent clinical trials [41,42,43]. It is safe to say that all three types of MSCs, listed in this review, demonstrate overall favorable safety profile while delivering major clinical benefits for the treated patients, battling osteochondral defects. Based on the data presented, it is apparent that MSC-based treatment shows potential in the treatment of patients classified as non-responders to current therapeutic options of RA/OA, or even to become the primary treatment for cartilage regeneration, steering away from the negative effects that come hand-in-hand with the traditional drug-based therapy [42]. Many questions regarding this novel therapeutic approach are still to be solved, for instance proper dosage, autologous vs. allogenic treatment, the underlying mechanism of MSC-related cartilage regeneration, etc. Another feature to be perfected is the development of a suitable delivery system for MSCs. Tissue engineering indicates that scaffolds could be useful for inducing cell adhesion, proliferation, and chondrogenic differentiation. Here we discussed two promising scaffold materials, the first one was a gradient hydrogel with the addition of nanosilicates, the second incorporated 3D printed porous metal scaffolds with infliximab-containing hydrogel. Both scaffolds were recognized to facilitate the necessary mechanical strength and biocompatibility, while upregulating the differentiation potential and viability of MSCs [44,78]. The infliximab-infused scaffold combined with ADMSCs was capable of down-regulation of inflammatory processes, replacement of damaged cartilage with new cartilage tissue, and subchondral bone repair induction in the RA rabbit model [44]. Next, we deliberated on osteochondral regeneration through implantation of scaffold-free 3D stem cell constructs, fabricated utilizing molds or via bio-3D printing. The results from studies implanting 3D scaffold-free constructs into animal models were capable of complete healing of the osteochondral defect at the cartilage level as well as the subchondral bone level [32,45]. For the future studies, there is a need for comparative studies to determine whether the scaffold-based or scaffold-free methods are more suitable for full osteochondral recovery in pre-clinical and later on clinical settings. Moreover, current progress reached in regenerative tissue engineering employing cell-based therapies provides evidence of immunomodulatory effect of MSCs, rather than the original hypothesis that implanted stem cells have the capacity to differentiate into native tissue [98]. Hence, exosome-based therapies and their application in osteochondral regeneration are currently being studied. The therapeutic potential of MSC-derived exosomes greatly encourages cartilage and subchondral bone repair, as revealed by studies in this report. Research groups claim that the therapeutic molecules contained in these extracellular vesicles support the regeneration of cartilage without adverse inflammatory response in animal models [26,46,47,49]. Nevertheless, it is essential to dissect the components present in MSC-exosomes and to explore the underlying mechanisms in cartilage repair. Moreover, efforts have been made to develop and establish a proper technique for the effective manufacturing of exosome-mediated products [26,49]. Still, it is necessary to seek separation/isolation techniques delivering high purity exosomes for clinical and commercialization purposes. Therapeutic approaches exploiting the combination of scaffolds and MSC-exosomes show a lot of promise for osteochondral defect repair scenarios. The “cell-free” tissue engineering strategies presented in this review, provide evidence that the exosome-delivery platforms, consisting of decellularized cartilage extracellular matrix scaffold, augment cartilage tissue regeneration through synergistic advantages of biological activation by exosome and structural support by scaffolding reservoir materials. They indicated that the implants promoted polarization of macrophages toward the M2 phenotype and inhibited inflammatory responses in the osteochondral region [50,51]. Additionally, a large number of questions regarding the regenerative processes remain to be answered. 

The studies described in this review suggest that it will be possible to establish a novel therapeutic approach with the help of mesenchymal stem cells and their derivates, that will override the limited efficacy of current treatments for osteochondral healing. However, the enigma behind in-depth mechanisms of endorsed regenerative mechanisms is still to be solved. Furthermore, additionally extensive research on safety, efficacy, and clinical relevance needs to be completed in the forthcoming years.
